# Supplementary material for: Quasi-Solid-State Polymer Electrolyte Based on Electrospun Polyacrylonitrile/Polysilsesquioxane Composite Nanofiber Membrane for High-Performance Lithium Batteries
Source: Materials (Basel). 2022 Oct 27;15(21):7527. doi: 10.3390/ma15217527 (PMC9658625; doi:10.3390/ma15217527)
Supplement: Supplementary file 1 [file materials-15-07527-s001.zip › materials-1965400-supplementary.pdf]

Quasi-Solid-State Polymer Electrolyte Based on Electrospun Polyacrylonitrile/Polysilsesquioxane Composite Nanofiber Membrane for High-Performance Lithium Batteries

Caiyuan Liu[1], Jiemei Hu[1], Yanan Zhu[1], Yonggang Yang[1], Yi Li [1,*], Qi-hui Wu [2,*]

[1] Jiangsu Key Laboratory of Advanced Functional Polymer Design and Application, Department of Polymer Science and Engineering, College of Chemistry, Chemical Engineering and Materials Science, Soochow University, Suzhou 215123, P.R. China.

[2] College of Marine Equipment and Mechanical Engineering, Xiamen Key Lab of Marine Corrosion and Smart Protective Materials, Jimei University, Xiamen 361021, P. R. China.

E-mail: Y. Li, liyi@suda.edu.cn; Qi-hui Wu, qihui.wu@jmu.edu.cn

Calculations

The electrolyte uptake was calculated based on the mass difference of membranes before and after sufficient immersion in the liquid electrolyte according to the following formula:

$$\text{Uptake (\%)} = \frac{W_w - W_d}{W_d} \times 100 \qquad \text{(S1)}$$

where W_d and W_w are the mass of the membrane before and after immersion in the liquid electrolyte, respectively.

Ionic conductivity (σ) was investigated by AC impedance analysis on a symmetrical cell of stainless SS/PSiO/SS steel, which can be further calculated by the formula:

$$\sigma = \frac{l}{R_b S} \quad \text{(S2)}$$

where l is the electrolyte membrane thickness, R_b stands for the electrolyte bulk resistance measured by EIS and S is the cross-sectional area. The activation energy of Li^+ conduction can be calculated by the Arrhenius equation:

$$\sigma = A\ exp(-\frac{E_a}{RT}) \quad \text{(S3)}$$

where A is the pre-exponential factor, E_a stands for the activation energy, T is the absolute temperature and R stands for the Boltzmann constant. The lithium-ion transference number (t_{Li^+}) of the IE at room temperature was determined by using chronoamperometry in combination with impedance spectroscopy measurements on a symmetrical battery of Li/IE/Li, the corresponding numerical results can be obtained by the formula

$$t_{\mathrm{Li}}^{+} = \frac{I_s R_b^0 \left(\Delta V - I_0 R_1^0\right)}{I_0 R_b^s \left(\Delta V - I_s R_1^s\right)} \quad \text{(S4)}$$

where I_0 and I_s are the initial and steady-state currents, ΔV is the polarization voltage (ΔV = 30 mV), R_b^0 and R_b^s are the bulk resistances (Ω) before and after polarization, R_1^0 and R_1^s are the interfacial resistances (Ω) before and after polarization, respectively.

Table S1. Summary of PAN- and polysilsesquioxane-based QSPE.

Materials	Fabrication methods	Electrolyte uptake (%)	Ionic conductivity (mS cm^{-1})	Capacity (mAh g^{-1})	Performance
PAN [1]	Electrospinning	1100	1.7×10^{-2} (20°C)	97.2 (0.05C)	High porosity and electrolyte uptake; low ionic conductivity and poor thermal stability
PAN/SiO_2 (27 wt%) [2]	Electrospinning	337	2.6 (25°C)	155 (0.2 C)	Superior electrochemical performance, good thermal stability
PAN/PBA (10 wt%) [3]	Electrospinning	487.1	2.7 (25°C)	152.2 (0.2C)	High electrolyte uptake, high ionic conductivity and good electrochemical stability
PAN/LATP (15 wt%) [4]	Electrospinning	525	3.6 (25°C)	150 (0.2C)	Low interfacial resistance with lithium metal
Polysilsesquioxane/ PMMA/PVDF [5]	Radical polymerization, Electrospinning	631.5	4.85 (25°C)	150 (0.2C)	High porosity, good thermal stability and excellent mechanical properties; complex preparation way
polysilsesquioxane [6]	Sol-gel preparation	900	2.1 (25°C)	111.6 (0.1C)	Good thermal stability, wide electrochemical window and ideal room temperature ionic conductivity; poor mechanical flexibility

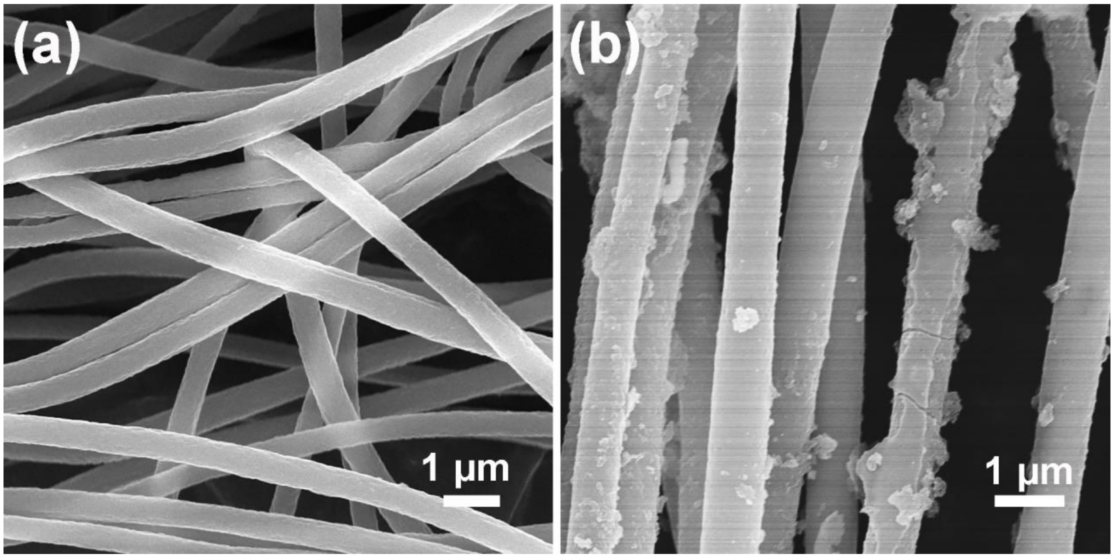

Figure S1. FE-SEM images of (a) PAN nanofibers and (b) PAN@PSiO composite nanofibers.

The formation mechanism of the PAN@PSiO nanofiber composite was as follows. According to the liquid crystal template mechanism first proposed by Mobil researchers, the cationic surfactant CTAB acted as the soft template. It formed supramolecular self-assembly via non-covalent bond interaction on the surfaces of PAN nanofibers, which acted as the hard template. BTEB acted as precursor. It hydrolyzed under the help of catalyst and adsorbed onto the surfaces of CTAB micelles by electrostatic interaction and they underwent synergistic self-assembly. After the polycondensation of BTEB (the sol-gel transcription) was completed, the core-shell structured composite nanofibers were fixed. The mesoporous polysilsesquioxane layer was obtained after the removal of CTAB micelles by extraction.

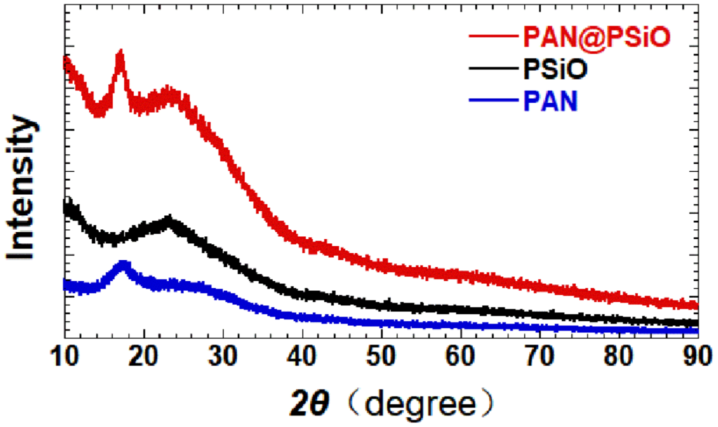

Figure S2. WAXRD patterns of PAN, PSiO and PAN@PSiO nanofibers.

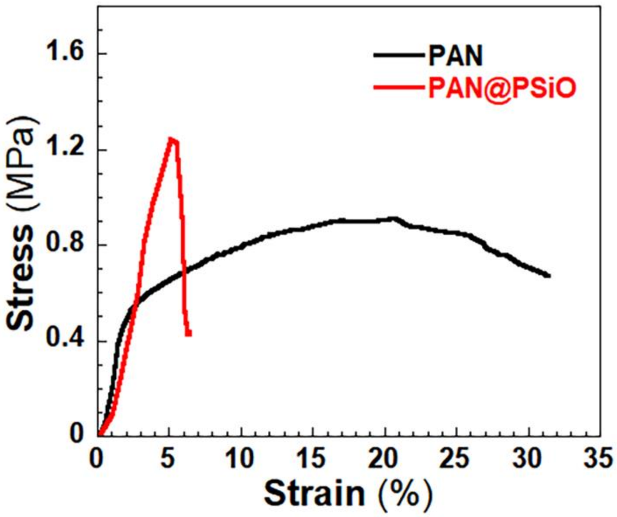

Figure S3. Stress-strain curves of PAN nanofiber membrane and PAN@PSiO composite nanofiber membrane.

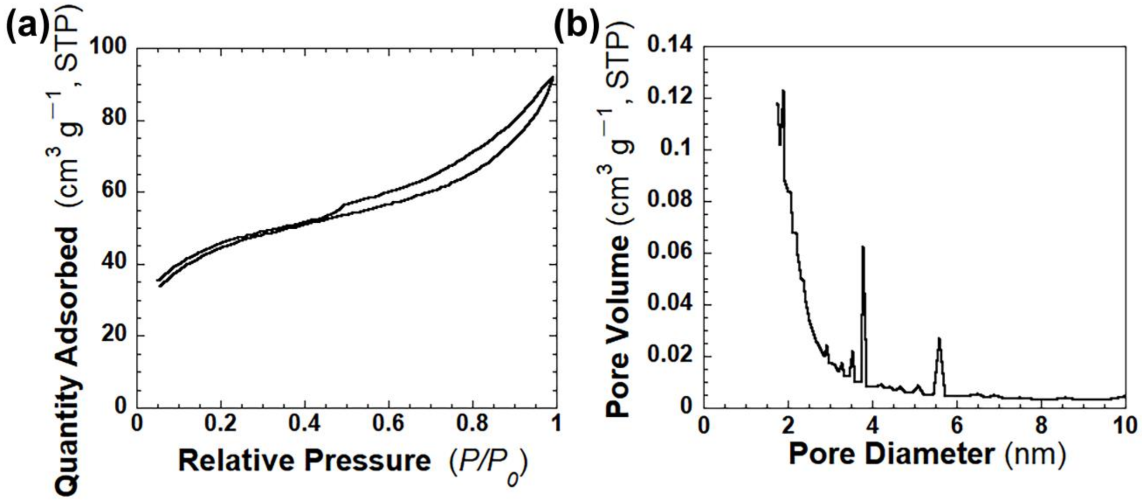

Figure S4. (a) Nitrogen sorption isotherms and (b) BJH pore size distribution plot calculated from the absorption branch for the PAN@PSiO composite nanofiber membrane.

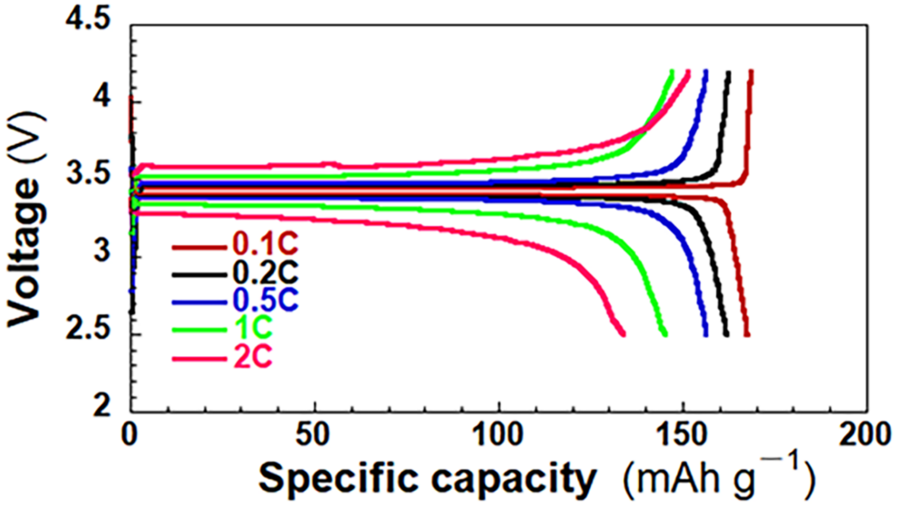

Figure S5. Initial charge and discharge curves of the $LiFePO_4$|PAN@PSiO|Li battery at current densities varied from 0.1C to 2C.

References

[1] P. Carol, P. Ramakrishnan, B. John, G. Cheruvally, Preparation and characterization of electrospun poly(acrylonitrile) fibrous membrane based gel polymer electrolytes for lithium-ion batteries, J. Power Sources 196 (2011) 10156.

[2] M. Yanilmaz, Y. Lu, J. Zhu, X. Zhang, Silica/polyacrylonitrile hybrid nanofiber membrane separators via sol-gel and electro-spinning techniques for lithium-ion batteries, J. Power Sources 313 (2016) 205.

[3] X. Yuan, A.A. Razzaq, Y. Chen, Y. Lian, X. Zhao, Y. Peng, Z. Deng, Polyacrylonitrile-based gel polymer electrolyte filled with Prussian blue for high-performance lithium polymer batteries, Chin. Chem. Lett. 32 (2021) 890.

[4] Y. Liang, Z. Lin, Y. Qiu, X. Zhang, Fabrication and characterization of LATP/PAN composite fiber-based lithium-ion battery separators. Electrochim. Acta 56 (2011) 6474.

[5] Y. Liu, X. Ma, K. Sun, K. Yang, F. Chen, Preparation and characterization of gel polymer electrolyte based on electrospun polyhedral oligomeric silsesquioxane-poly(methylmethacrylate)/polyvinylidene fluoride hybrid nanofiber membranes for lithium-ion batteries, J. Solid State Electrochem. 22 (2018) 581.

[6] J. Hu, Y. Zhu, C. Liu, Y. Yang, Y. Li, Quasi-solid-state electrolyte membranes based on helical mesoporous polysilsesquiox-ane nanofibers for high-performance lithium batteries, J. Taiwan Inst. Chem. Eng. 135 (2022) 104399.
